# Lack of association between cluster headache and *PER3* clock gene polymorphism

**DOI:** 10.1186/s10194-016-0611-3

**Published:** 2016-02-29

**Authors:** Hilde K. Ofte, Erling Tronvik, Karl B. Alstadhaug

**Affiliations:** Department of Neurology, Nordland Hospital Trust, 8092 Bodø, Norway; Department of Neuroscience, Norwegian University of Science and Technology, Edvard Griegs gate 8, 7491 Trondheim, Norway; St.Olavs Hospital, Trondheim, Norway; The Arctic University of Norway, Tromsø, Norway

**Keywords:** Cluster headache, Chronotype, PERIOD3, Clock genes, Shift work, Horne-Ostberg Morningness eveningness questionnaire

## Abstract

**Background:**

Cluster headache (CH) is regarded as a chronobiological disorder. The hypothalamic biological clock may thus be involved in the pathophysiology, but few studies have actually investigated this in CH patients. A variable number tandem repeat (VNTR) polymorphism of the *PER3* clock gene has been associated to preferred daily rhythm (chronotype) in several studies. We aimed to study the distribution of *PER3* VNTR polymorphisms and chronotypes in a CH population.

**Methods:**

We used blood samples from a biobank of CH patients for genetic tests, and invited all tested patients to complete the Horne-Ostberg Morningness-eveningness Questionnaire (MEQ), the Pittsburgh sleep quality Index (PSQI) and the Shift Work Index. Genotypes were compared to a previously tested population of 432 healthy students.

**Results:**

One hundred forty nine patients were genotyped, and we found no difference in *PER3* VNTR polymorphisms between patients and controls. Seventy-four patients completed the MEQ (54 men, 20 women, mean age 52.3 years ± 13.4), and chronotypes were as follows: 12 % morning-, 37 % intermediate-, and 51 % evening types. Compared with a previous Danish study of CH patients and controls, there were no difference in chronotype distribution. Sixty percent of patients were defined as bad sleepers (PSQI >5), and 51 % of patients currently employed were shift workers.

**Conclusions:**

No association between CH, *PER3* VNTR polymorphism and chronotype was found in this study.

## Background

Cluster headache (CH) is a periodic disorder, with a striking rhythmic occurrence of headache attacks and bouts. Up to 80 % of patients report a predictable diurnal rhythmicity of attacks [1], and the attacks seem to occur most often at night between midnight and 04 a.m. [2], waking the patient from sleep. CH has been associated with shift work and chronic insomnia [2], and it has recently been demonstrated that CH patients in bouts have reduced REM sleep and fewer sleep arousals compared to controls [3]. The periodicity of attacks and apparent sleep disturbances imply that CH is a chronobiological disorder, and that the hypothalamic biological clock (the suprachiasmatic nucleus, SCN) may be involved in CH pathophysiology [4].

The SCN governs the biological rhythms of the body, oscillating in a strong 24-h cycle caused by transcription-translation loops of the so-called clock genes [5]. One of the key transcriptional loops running the clock is the CLOCK:BMAL1 cycle, where the *CLOCK* and *BMAL1* gene proteins join to form a complex that initiates transcription of the *PER* (*PER1*,*2* and *3*) and *CRY* (*CRY1* and *2*) gene families [6]. The protein products of these genes translocate to the nucleus and inhibit further transcription of the CLOCK:BMAL1 complex, thereby inhibiting their own transcription. *CK1δ/ε* gene coded kinases phosphorylate and degrade the PER/CRY proteins, allowing the cycle to start over again. The length of the cycle varies between individuals and sets the period of the biological clock.

The internal clock cycle is reset every day by several external factors, of which light is the most important. Individual differences in the endogenous rhythms, in addition to sensitivity to light and sleep homeostasis, determine the biological basis to what we in daily life recognize as individual differences in diurnal preference (chronotype). Morning types (larks) seem to have shorter circadian cycles than evening types (night owls), and advanced sleep phase disorder (ASPD) and delayed sleep phase disorder (DSPD) are viewed as extremes in opposite ends of the continuum of diurnal preferences [7]. A variable number tandem repeat (VNTR, Table 1) of the DNA in the clock gene *PERIOD3* (*PER3*, rs57875989) has been shown to correlate with diurnal preference in humans [8], and the short allele of the *PER3* VNTR was associated to DSPD in two studies [9, 10].

**Table 1:** Variable number tandem repeats

A variable number tandem repeat occurs in DNA when a pattern of one or more nucleotides is repeated, and the repetitions are directly adjacent to each other. In the human PER3 gene, the short allele contains four tandem 54-bp repeats, and the long allele has five such repeats. As each individual has two sets of alleles, this produces three possible genotypes: *PER3* 4/4, 4/5 and 5/5.	

A mismatch between the internal clock cycle and external time cues causes *circadian misalignment*, which over time may lead to various health problems [11]. Both chronotype and clock genotype contribute to how well individuals adapt to such misalignment [12, 13]. If cluster headache is indeed a chronobiological disorder, an important question is whether the pathology resides in the biological clock itself, or in the adaptation between the clock and the environment. We aimed to study the *PER3* VNTR distribution, chronotype and prevalence of sleep disturbances in a CH population, to evaluate if a specific genotype is associated with a higher risk of CH.

## Methods

### *PER3* VNTR genotyping

We used blood specimens obtained from a biobank located at St. Olavs Hospital in Trondheim. The biobank is founded by the Norwegian Advisory Unit on Headaches, and contains blood samples from headache patients living in the middle and northern part of Norway. All patients included in the biobank are consulted by a headache specialist, validating the headache diagnosis according to the International Classification of Headache Disorders (ICHD, version 2 and 3-beta) [14]. In addition, the patients must complete a form with questions regarding clinical characteristics, use of medication and work status. In spring 2014, all patients registered in the biobank with a diagnosis of CH were identified and DNA extracted from the specimens. There was no kinship between the patients. DNA samples were purified, and polymerase chain reaction (PCR) amplification of the *PER3* polymorphism performed according to previously validated methods [9, 15]. We used forward primer 5’-CAAAATTTTATGACACTACCAGAATGGCTGAC-3’ and reverse primer 5’-AACCTTGTACTTCCACATCAGTGCCTGG-3’, as reported by Ebisawa et al. [9]. Gel electrophoresis was used to differentiate the *PER3* genotypes. We used information on clincial characteristics from the biobank to compare the different genotypes.

The *PER3* genotype distribution in the CH population was compared to that of a previously published population of 432 healthy Norwegian students (176 males and 256 females, mean age 22 years, SD ± 2,6) [15].

### Chronotyping and sleep assessment

All the genotyped patients subsequently received an invitation by mail to participate in further studies, and were asked to complete and return the Horne-Ostberg Morningness-Eveningness Questionnaire (MEQ), Pittsburgh Sleep Quality Index (PSQI), and the Standard Shiftwork Index (SWI). All three questionnaires are validated in Norwegian translations.

#### Horne-Ostberg morningness-eveningness questionnaire (MEQ)

The MEQ is one of the most widely used and best validated tools for measuring diurnal preference [16]. It consists of 19 multiple-choice items, producing a score ranging from 16 to 86, with high scores indicating morning preference and low scores indicating evening preference. The initial chronotype cut-off points were validated for a population of young students, and as chronotype is strongly influenced by age and social factors, validated cut-off points for a middle-aged, non-shift working population have later been made [17]. We had no control group of our own for these data, but compared the results with a recent Danish study, chronotyping 275 CH patients and 145 controls [1]. The authors kindly provided chronotype distribution in the Danish populations adjusted for age (private correspondence).

#### Pittsburgh sleep quality index (PSQI)

The PSQI is a validated, retrospective measure of subjective sleep quality and disturbances during the last month [18]. It consists of 19 items grouped into seven equally weighted component scores, with higher scores indicating poorer sleep quality. The maximum score is 21, and a score above five indicates a poor sleeper.

#### Standard shiftwork index (SWI)

The SWI is a standardized battery of questions developed to study the psychological and physiological impact of shift work [19]. We used it mainly to identify shift workers, as the small number of participants made detailed studies on adaptation to different shifts less reliable.

### Statistical methods, power calculation and ethics

The null hypothesis was that there is no difference in *PER3* polymorphism or chronotype distribution between CH patients and controls. We also hypothesized that in the subgroup analysis; there would be no correlation between *PER3* genotype and MEQ-score, *PER3* genotype and PSQI-score, and between the MEQ and PSQI. The frequencies of genotypes and chronotypes were compared with chi-square tests. Continuous variables were compared between genotypes with one-way ANOVA. The association between genotype and MEQ or PSQI scores was tested in a multiple, linear regression model, adjusted for age, gender and shift work. The level of significance was set at 0.05 for all statistical tests. No a priori power calculations were made.

The data were analyzed using the SPSS software package for Windows version 21 (SPSS Inc, Chicago, IL, USA). The Research Ethics Committee of Northern Norway reviewed the protocol and recommended the study. The Norwegian Data Protection Authority approved of data registration.

## Results

### *PER3* VNTR genotyping

Genetic analysis of *PER3* VNTR was performed in 149 CH patients (109 males and 40 females, mean age 54.1 years, SD ± 14.0). Table 2 shows the distribution of genotypes in 432 healthy controls, the total group of 149 CH patients, and in the subgroup of 74 CH patients who were also chronotyped. When comparing the total CH population to healthy controls, there was no difference in *PER3* VNTR polymorphism (*χ*^*2*^ (2) = 0.016, *p* = 0.992). The genotype distribution was in Hardy-Weinberg equilibrium, and the *PER3* allele frequencies were the same for both populations (0.67 for the 4-allele and 0.33 for the 5-allele). Table 3 shows clinical characteristics in the CH population, divided between the *PER3* genotypes. There were no statistically significant differences between the three genotypes with regards to gender, age or clinical characteristics (calculations not shown). Unfortunately, there was no information available regarding episodic or chronic CH.

**Table 2:** *PER3* genotype distribution in 432 healthy controls and the total group of 149 CH patients, and *PER3* genotype distribution, MEQ and PSQI scores in the subgroup of 74 patients who completed both questionnaires

PER3 genotype	Controls	CH patients	CH patients	MEQ^a^	PSQI^a^
			Subgroup^a^	(Mean ± SD)	(Mean ± SD)
4/4	192 (44.5 %)	67 (45.0 %)	32 (43.2 %)	52.5 ± 9.8	9.1 ± 4.8
4/5	191 (44.0 %)	65 (43.5 %)	32 (43.2 %)	51.0 ± 11.7	8.1 ± 4.6
5/5	49 (11.5 %)	17 (11.5 %)	10 (13.5 %)	49.5 ± 8.9	6.5 ± 4.1
Total/mean	432	149	74	51.4 ± 10.5	8.4 ± 4.6

^a^Patients included in MEQ and PSQI analysis, *n* = 74

**Table 3:** Clinical characteristics of 149 genotyped CH patients, divided between the three *PER3* genotypes 4/4, 4/5 and 5/5

PER3 genotype	4/4	4/5	5/5
Gender (M/F)	50/17	50/15	9/8
Age at inclusion (years)	52.8, SD ± 14.5	55.6, SD ± 13.5	53.9, SD ± 14.5
Comorbid migraine without aura (N)	1 (1.5 %)	0	3 (17.6 %)
Comorbid migraine with aura (N)	5 (7.5 %)	5 (7.7 %)	1 (5.9 %)
Comorbid tension type headache (N)	1 (1.5 %)	1 (1.5 %)	0
Age at onset (years)	28.9, SD ± 13.1	31.0, SD ± 14.5	31.1, SD ± 13.9
Headache intensity: strong (N)	9 (13.4 %)	4 (6.2 %)	1 (11.8 %)
Headache intensity: extra strong (N)	37 (55.2 %)	39 (60 %)	10 (58.8 %)
Attack duration (minutes)	13.9, SD ± 8.5	13.9, SD ± 9.1	19.0, SD ±11.0
Number of attacks per month	5.7, SD ± 8.2	4.0, SD ± 7.6	4.7, SD ± 11.2
Number of attacks per day	3.3, SD ± 2.2	3.6, SD ± 3.2	3.0, SD ± 2.1
Total number of headache days in the last 3 months	12.3, SD ± 12.1	12.3, SD ± 11.0	17.1, SD ± 16.6
Total number of patients	*N* = 67	*N* = 65	*N* = 17

### Subgroup analysis

Eighty of the 149 CH patients responded to our invitation and returned the questionnaires. Of these, 74 completed both the MEQ and PSQI forms and were included in subgroup analysis (54 men and 20 women, mean age 52.3 years ± 13.4). The mean MEQ was 51.4 ± 10.5, and based on individual values we found the following chronotype distribution: 38 (51 %) evening type, 27 (37 %) intermediate type, and nine (12 %) morning type. Table 4 shows chronotype distribution in the present CH patient subgroup, compared to the previously chronotyped CH population and healthy controls from Denmark [1]. There was no difference in chronotype distribution between the groups (*p* = 0.595).

**Table 4:** Chronotype distribution in the present cohort of 74 CH patients in percent, as well as chronotypes described in a Danish CH population and their healthy controls

	Norwegian CH patients (%)	Danish CH patients (%)	Danish controls (%)
Evening type (MEQ scores 16–52)	51	45	42
Neither type (MEQ scores 53–64)	37	43	41
Morning type (MEQ scores 65–86)	12	12	17
Total number (N)	74	275	145

MEQ score cut-offs from Taillard [17] are used

The mean PSQI was 8.4 ± 4.6, and 44 of the responders (60 %) were defined as bad sleepers (PSQI > 5). Table 2 shows mean MEQ and PSQI in each *PER3* genotype. Seventy-six patients answered questions about shift work. Of the 49 patients currently employed, 25 (51 %) were shift workers and another nine (18 %) had previously worked shifts. Of the total group of 76 responders, 40 (52 %) were current or previous shift workers.

We found no association between *PER3* VNTR polymorphism and chronotype (*χ*^*2*^(4) = 4.42, *p* = 0.352). The multiple regression analysis adjusted for age, gender and shift work, showed no association between *PER3* polymorphism and MEQ score. The PSQI scores seemed to decline from the *PER3* 4/4 to the *PER3* 5/5 genotype, but this was not statistically significant. Likewise, there was no association between MEQ and PSQI scores.

## Discussion

We found no association between *PER3* VNTR polymorphism and CH in his study. There seem to be a high proportion of evening types in this Norwegian CH cohort, but we found no statistically significant difference in chronotype distribution when compared to a previously published Danish CH population and their healthy controls. The lack of association may be due to small sample size, or to the fact that MEQ is heavily influenced by age and social factors such as work schedules [20]. Thus, the activities of daily life may mask a true difference in clock period between individuals.

To the best of our knowledge, only one clock gene mutation has previously been investigated in a CH population. A single nucleotide polymorphism (SNP) in the downstream region of the *Clock* gene (T3111C) was studied in three separate Italian studies [21–23], all of which found no difference in genotype distribution between CH patients and healthy controls. The complexity of the biological clock was demonstrated in a genome-wide study from 2009, in which screening of small interfering RNAs showed that more than 200 genes were involved in clock functioning, regulating amplitude and period [24]. This illustrates that further research is needed to determine the role of clock involvement in CH.

Sixty percent of the CH patients in our study were defined as bad sleepers, and sleep disturbances seem to be a consistent finding in studies of CH [1, 2, 4]. A mean PSQI of 8.4 is high even when compared to other headache populations. In a study from 2009, the authors found a mean PSQI of 5.9 ± 3.0 in migraine patients who suffered ≥ 8 migraine days a month [25]. Sleep is one of the most important attack triggers in CH [2], but insomnia is not merely a result of nocturnal headache disrupting sleep, as sleep quality remains poorer compared to controls as long as one year after last headache attack [1]. Recent studies have shown that sleep homeostasis and the circadian oscillations of the SCN are more interconnected at the molecular level than previously thought [26], and the sleep disturbances in CH populations could therefore be a direct result of clock involvement in CH pathophysiology. The relationship between CH and sleep is further underlined by the association between CH and a missense SNP in the hypocretin receptor-2 gene (HRCTR2) reported in several studies, although a recent meta-analysis cast doubt on this finding [27].

Fifty-one percent of the patients in this cohort currently employed were shift workers. For comparison, about 33 % of the general workforce in Norway work outside regular office hours (https://www.ssb.no/en/arbeid-og-lonn/statistikker/yrkeaku/aar/2012-04-26#content). Adaptation to shift work is influenced by both chronotype and clock genotype [12]. Night workers are more probable to be definite evening types compared to daytime workers [20], and it seems that morning types adapt better to daytime work than evening types. The CH patients in our study are mostly evening types, and we do not know if their evening preference is a result of the shift work or if they choose to work shifts because it suits them better. Theoretically, shift work may result in a circadian misalignment that could trigger CH in predisposed individuals. Further research is needed on the association between CH and shift work occupation.

## Strengths and limitations

On the strong side, headache specialists had validated the CH diagnosis of all the participants prior to inclusion in the headache biobank. CH patients and controls were mainly ethnic Norwegians, thus suitable for genetic comparison. The controls were significantly younger than the CH patients, and there were more men in the patient group, but as genotype is a constant trait, and not associated with reduced lifetime expectancy or to a specific gender, this is irrelevant for our analysis. We used previously validated genetic tests and test tools, as well as validated Norwegian translations of the questionnaires.

The greatest limitation to our study concerns the issue of statistical power. Power calculations in genetic association studies rely heavily on the effect size that the candidate gene exerts on the disease or trait studied. This effect is unknown in our case, and could only be based on assumptions. It is generally accepted that common complex diseases are unlikely to be caused by a single locus of large effect, and that genotypic relative risks are likely to be in the range of 1–1.5 [28]. CH is a rare disease, but it is most probably of a complex genetic origin. The limited access to patients makes large genetic studies on CH difficult to conduct, and our study is probably underpowered. This warrants caution in interpreting the results. However, genetic studies are important to develop a better understanding of the pathophysiology even in rare headache disorders. Despite its small sample size, we believe that this study adds to the knowledge of CH pathophysiology.

## Conclusion

This study shows that CH patients have the same distribution of *PER3* VNTR polymorphism and chronotype as healthy controls. There is a high frequency of sleep disturbances and shift work occupation in the CH population. Further research on the association between CH, clock genes and their regulators are needed.
